# Comparative Clinical Study of Bactigras and Telfa AMD for Skin Graft Donor-Site Dressing

**DOI:** 10.3390/ijms12085031

**Published:** 2011-08-08

**Authors:** Pornprom Muangman, Sooksan Nitimonton, Pornanong Aramwit

**Affiliations:** 1 Burn Unit, Trauma Division, Department of Surgery, Faculty of Medicine Siriraj Hospital, Mahidol University, Bangkok 10700, Thailand; E-Mails: pmuangman@yahoo.com (P.M.); ss79ntmt@yahoo.com (S.N.); 2 Department of Pharmacy Practice, Faculty of Pharmaceutical Sciences, Chulalongkorn University, Bangkok 10330, Thailand

**Keywords:** Bactigras, burn, chlorhexidine, donor site wound, polyhexamethylene biguanide, Telfa AMD

## Abstract

The Bactigras^®^ paraffin tulle coated with chlorhexidine is normally used for the treatment of donor-site wounds in burn patients who received split-thickness skin grafts in several centers. It has some disadvantages, such as adhesion to wound surfaces and pain from the irritation caused by this dressing. The Telfa AMD^®^, a non-adherent wound dressing which consists of absorbent cotton fibers impregnated with polyhexamethylene biguanide enclosed in a sleeve of thermoplastic polymers, is a new option for donor-site wound care which causes less adherence to the wound. The purpose of this study was to compare clinical efficacy of these two dressings for the management of donor-site wounds. Thirty-two patients who received split-thickness skin grafts by donor site harvesting from the thigh were enrolled in this study and randomized into two groups receiving either the Bactigras^®^ or the Telfa AMD^®^ wound treatment. Re-epithelialization, pain, infection and cost-effectiveness analyses were compared between both groups. The results showed that there was no significant difference in age, area of donor sites or length of hospital stays between the groups (*p* > 0.05). However, the day of re-epithelialization (≥90%) was significantly shorter in patients treated with the Telfa AMD^®^ compared to the Bactigras^®^ group (14.00 ± 3.05 *vs.* 9.25 ± 1.88 days for Bactigras^®^ and Telfa AMD^®^ groups, respectively, *p* < 0.001). The average pain score was also significantly lower in the Telfa AMD^®^ group (1.57 ± 0.55 *vs.* 4.70 ± 1.16, *p* < 0.001). There was no difference in the cost of treatment between the groups (4.64 ± 1.97 *vs.* 5.72 ± 2.54 USD, *p* = 0.19). This study indicated that the Telfa AMD^®^ was an effective dressing for the treatment of donor-site wounds.

## 1. Introduction

Split-thickness skin grafting is the most frequently used procedure in plastic surgery for the replacement of damaged or missing skin. The success of the procedure depends on the complete integration of the graft with the recipient bed and on the re-epithelialization of the skin graft donor site [1–3]. Treatment of the split-thickness autograft donor sites has been studied over the years but there is no standard treatment for managing these sites. The treatment protocol involves a variety of techniques and dressing materials, and all of them aim for a fast, spontaneous re-epithelialization of the donor sites [4]. Adequate wound treatment aims to prevent or reduce the risk of associated complications and to facilitate the healing process whilst considering the patients’ physical and mental well-being during the treatment process [5]. The ideal treatment method protects the wounds from dehydration and mechanical trauma, prevents infection and reduces re-epithelialization time, and provides maximum comfort for the patient [6].

In general, the methods of treating donor wounds are categorized as open, semi-open, and closed [7]. The open method refers to the method where the wound remains exposed and it is allowed to heal without a dressing. The semi-open method means the wound bed is covered with dressing just once and then the wound is allowed to heal by the open method while in the closed method, the wound dressing is left intact for two to seven days. The most common approach is to make multiple dressing changes until the wound is completely healed. Among these techniques, the closed methods meet these requirements for adequate wound treatment to a large extent and have become the most attractive technique over the last decade [8,9]. Paraffin gauze dressing is recognized as a standard treatment for split-thickness skin graft donor sites [10]. It is considered to be non-adherent; nevertheless, it usually sticks to the wound surface while it absorbs exudate. Early removal of the dressing may lead to skin maceration or wound infection and wound epithelialization may slough off, accompanied by local pain aggravation and wound deepening [11].

Chlorhexidine is an antibacterial agent which is effective against a wide range of Gram-positive and Gram-negative bacteria, including Methicillin-resistant *Staphylococcus aureus* (MRSA). Traditionally, mesh paraffin gauze with chlorhexidine is normally used for the treatment of donor-site wounds. Chlorhexidine can bind to bacterial cell walls at low concentrations, causing an alteration of the bacterial cell osmotic equilibrium and leakage. One of the disadvantages of this traditional gauze includes adherence to wounds, which can cause trauma to epithelial cells when removed. The Telfa AMD^®^, a non-adherent wound dressing, consists of a thin layer of absorbent cotton fibers impregnated with polyhexamethylene biguanide (PHMB), enclosed in a sleeve of poly (ethylene terephthalate), a thermoplastic polymer, that is perforated in a regular pattern and sealed along two edges [12,13]. Polyhexamethylene biguanide is a polymeric biguanide with a broad antimicrobial spectrum against both Gram-positive and Gram-negative bacteria, fungi and yeasts [14,15]. It has been used for several years as an antiseptic agent in medicine [16]. Polyhexamethylene biguanide binds to the surfaces of organisms causing instability and extensive disruption of their cytoplasmic membranes. It has a low systemic toxicity and poor absorption through skin. Since infection is another factor which retards wound healing, a broad spectrum antimicrobial agent may be beneficial for wound treatment as well as for split-thickness skin graft donor sites.

The purpose of this study was to compare the clinical efficacy of the PHMB-containing wound dressing in the thermoplastic polymer with the paraffin tulle dressing coated with chlorhexidine, the standard treatment for skin graft donor sites in the management of donor-site wounds.

## 2. Result and Discussion

The alternative dressing materials used for split-thickness skin graft donor sites present differences in healing times, infection and patient comfort. Since all of the dressings possess some unique properties, no ideal dressing is available on the market [17]. The challenge in managing donor site wounds is to promote healing as quickly as possible while minimizing adverse effects and complications [10]. If a complication such as infection occurs, the split-thickness defect may convert into a full-thickness loss, analogous to a third-degree burn [10]. Because of this, material which contains antibacterial agents should be applied; however, it should not hinder wound healing and, in addition, it should preferably have a promoting effect on epidermal healing [18]. In contrast to the great number of studies in which different techniques for the dressing of donor sites were evaluated, our present study compared the efficacy of two occlusive dressings, the Bactigras^®^ dressing and the Telfa AMD^®^. The Bactigras^®^ dressing is commonly used as a standard protocol of donor site wound dressing at our institute, which is similar to many burn centers using paraffin tulles coated with chlorhexidine [10]. The Telfa AMD^®^ is commercially available as a non-adherence dressing with antimicrobial agents, which may have advantages in terms of patient comfort.

**Figure 1.**
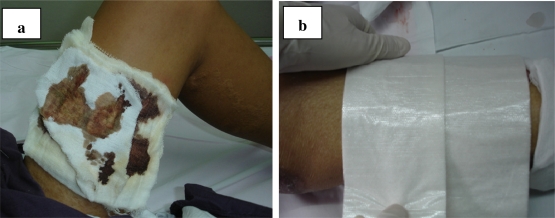
New donor sites treated with Bactigras^®^ (**a**) and Telfa AMD^®^ (**b**), respectively.

Thirty-two patients were enrolled in this study and all completed the follow-up period. Before the operation, all subjects were randomized into the two treatment arms. There were no significant differences in demographic parameters between the two groups at baseline including age, area of donor site and length of hospital stay. However, there was a significant difference in gender between both groups (Table 1). During the study period, clinical observations showed that both dressings were easily applied and did not require special supplies. The adhesion of the Telfa AMD^®^ was lower than that of the Bactigras^®^ dressing, and after moisturizing the Telfa AMD^®^ was easily removed without damaging the newly formed epithelium. However, the Bactigras^®^ dressing had a greater level of adhesion to the wound surfaces, and there was a risk of damaging the delicate epithelium. Figure 1 shows the new donor sites to be treated with the Bactigras^®^ (Figure 1a) and Telfa AMD^®^ (Figure 1b) dressings.

The donor sites treated with the Telfa AMD^®^ had a shorter re-epithelialization time and the length of time taken for more than 90% re-epithelialization was significantly different when compared to that of the patients in the Bactigras^®^ group (Table 2).

The pain assessment also showed a statistically significant difference when comparing the applications of Telfa AMD^®^ and the Bactigras^®^ dressing. The average grade of pain in the Telfa AMD^®^ applications was much lower on all evaluation days (Table 3). However, the average grades of pain in both groups were within the ranges of discomfort and very slight pain, not within the range of real pain, which requires analgesia.

Our results indicated that overall wound healing, as measured by the percentage of epithelialized dermis, was faster with the Telfa AMD^®^ than with the Bactigras^®^ dressing. The faster re-epithelialization rate observed with the Telfa AMD^®^ can partially be explained by its physical properties since it contains poly (ethylene terephthalate) polymers. Since the Telfa AMD^®^ has lower adhesion properties, it not only prevents trauma to the new and delicate epithelium during dressing removal, but it also provides a good moist environment, which is preferred for epithelial cell proliferation and migration [19]. This concept was well supported by evidence from many previous studies which showed faster re-epithelialization rates when moist-environment dressings were compared with traditional dry dressings [7,19–21].

On the other hand, the Bactigras^®^ dressing has a greater absorptive effect, which resulted in a greater amount of adhesion. During dressing removal or patient movement, it is possible to damage the delicate epithelial cells which can slow wound re-epithelialization as well as increase patient discomfort. Therefore, the physical difference between these two dressings may be the reasons for our results. Moreover, a case of *Pseudomonas aeruginosa* infection, a bacterium which is of especial concern in patients with burns, was found in a patient treated with the Bactigras^®^ dressing, which indicated that PHMB might be more beneficial in infection control than chlorhexidine. However, no mortality or any side effects from either of the dressings occurred in this study. With respect to bacterial growth, one patient in the Bactigras^®^ group was found to have a local infection on the tenth day with *P. aeruginosa* (10^2^), and no local infection was observed in the Telfa AMD^®^ group. However, the microbial numbers were below the critical values. After receiving a standard systemic antibiotic, no isolated bacteria were found on the next day.

Pain is the main cause of patient discomfort which challenges burn treatment protocols. Our results indicated that the Telfa AMD^®^ results in statistically less pain at the donor site from the first day of treatment, and the pain score was significantly reduced after three days. Even though the pain score has completely subjective characteristics, it is a reflection of how comfortable patients feel during treatment and it has been widely used in similar studies.

The results from the comparative cost-effective analysis showed that the cost difference between both dressings was insignificant. The total cost of patients treated with the Telfa AMD^®^ was slightly higher than the Bactigras^®^ dressing, which may have been due to the cost of the dressing itself.

## 3. Experimental Section

This was a prospective, randomized control study comprising 32 patients treated at the Siriraj Burn Unit, Thailand during December 2008–February 2010. It was designed to be open-labeled and observer blinded. The study was approved by Institutional Review Board Committee of the hospital, and written informed consent was obtained from each patient who enrolled in the study.

Twenty-two of the patients were men and ten were women, aged 16–78 years old. All monitored patients had similar burns with regard to the burn area and the depth of donor sites. Patients who need skin graft operation were randomized by computer and placed into two groups: 16 donor sites were treated with the Bactigras^®^ paraffin tulle dressing coated with chlorhexidine (Smith & Nephew Healthcare Limited, Hull, UK), and 16 donor sites were treated with the Telfa AMD^®^ cotton fiber impregnated with PHMB (Covidien, Mansfield, MA, USA). The demographic were collected from each subject in both group including age, gender, area of donor site (cm^2^), operative time and length of hospital stay.

The donor site was at the proximal thigh area. The patients were excluded if they were allergic to paraffin, chlorhexidine, poly (ethylene terephthalate) or PHMB. They were also excluded if there were lesions on both thighs, if they had psychiatric problems or multiple injuries (more than two systems involved), if they were immunocompromised, such as with renal failure, cirrhosis or malnutrition, and if they were receiving radiation or chemotherapy for malignancy. Patients with diabetes mellitus, systemic lupus erythematosus or other connective tissue diseases, and patients who had donor sites in areas other than the thigh were also excluded. Patients who did not comply with the study protocol, or who had a skin graft which had previously been harvested from the same donor site area, could not be involved in this study either.

All of the skin grafts (0.010 inches thickness) were taken from the thigh using a Zimmer^®^ Air Dermatome Skin Grafting System (Zimmer, Ltd., Swindon, UK). The area of donor site (cm^2^) was calculated using Image J Java-based image processing program developed by the National Institutes of Heath. Immediately after harvest, the donor site was covered with a saline-soaked gauze for hemostasis until surgery was completed. The Bactigras^®^ or Telfa AMD^®^ dressing were applied to the donor wound covering about 1 cm of intact skin. The dressing was secured by a sterile gauze. The donor site wounds were inspected every day after operation. None of the dressings were changed until the wounds were completely dry and the dressings fell off.

A donor site follow-up chart was used to conduct the clinical follow-up of the healing process. The information gathered in the chart included the percentage of re-epithelialization of each donor site area, the state of healthy skin on the periphery of each donor site and local signs of infection. Moreover, local pain was also followed-up using a visual analogue pain scale from 0 (no pain) to 10 (maximal severe pain) which was recorded at 30 minutes after the open wound, then at days 3, 7, 14 and 21 or when the dressing fell off. Normal saline solution was used to moisturize the dressing prior to removal and it is considered as neutral solution, no interaction has been found between normal saline and all dressing materials. The patients and the observer were blinded to the type of dressing in each donor site.

Infection was also evaluated by swab cultures for a microbiological analysis which was performed routinely once a week on Tuesday. A cost-effectiveness analysis was also compared between these treatment groups. The costs of the dressings, supplies and nursing labor were used to calculate the treatment cost.

Comparative analyses of the patients in both groups were performed using two-tailed unpaired student’s *t*-test with SPSS version 10.0 (SPSS Inc., Chicago, IL, USA). The results were expressed as mean (±SD). A *p* < 0.05 was considered as statistically significant.

## 4. Conclusions

Both the Bactigras^®^ dressing and the Telfa AMD^®^ are easy to apply in clinical practice. They can both protect wounds against mechanical trauma and provide comfort for the patients. However, the Telfa AMD^®^ provides a shorter re-epithelialization time, prevents infection and generates lower pain level in comparison with the Bactigras^®^ dressing. The treatment cost difference between these two dressings is negligible.

## Figures and Tables

**Table 1.** Demographics data of patients in each group.

**Demographics Data**	**Paraffin Tulle + Chlorhexidine Dressing (Bactigras^®^) (Range)**	**Cotton Fiber + PHMB (Telfa AMD^®^) (Range)**	***p*-Value**
Gender (male:female)	14:2	8:8	0.02 *
Age (years)	36.19 ± 19.81 (16–78)	29.13 ± 12.55 (17–50)	0.24
Area of donor sites (cm^2^)	1,016.38 ± 498.56 (336–2,340)	935.83 ± 436.47 (270–2,052)	0.63
Length of hospital stays (days)	53.63 ± 36.22 (9–126)	47.69 ± 31.30 (13–124)	0.62

* indicates significant difference (*p* < 0.05).

**Table 2.** Efficacy of Bactigras^®^ and Telfa AMD^®^ in donor site wounds.

	**Paraffin Tulle + Chlorhexidine Dressing (Bactigras^®^) (Range)**	**Cotton Fiber + PHMB (Telfa AMD^®^) (Range)**	***p*-Value**
Day of reepithelization (≥90%)	14.00 ± 3.05 (9–21)	9.25 ± 1.88 (7–13)	<0.001 *
Pain score	4.70 ± 1.16 (2.20–6.64	1.57 ± 0.55 (0.57–2.57)	<0.001 *
Number of infection site	1	0	–
Cost of treatment (USD)	4.64 ± 1.97 (2.12–9.55)	5.72 ± 2.54 (1.73–12.09)	0.19

* indicates significant difference (*p* < 0.05).

**Table 3.** Average grade for the assessment of the pain in treatment with Bactigras^®^ and Telfa AMD^®^.

**Pain Score**	**Paraffin Tulle + Chlorhexidine Dressing (Bactigras^®^) (Range)**	**Cotton Fiber + PHMB (Telfa AMD^®^) (Range)**	***p*-Value**
First day	6.81 ± 1.17 (5–9)	2.56 ± 1.41 (0–5)	<0.001 *
Third day	6.38 ± 1.45 (4–9)	1.88 ± 1.20 (0–4)	<0.001 *
Seventh day	5.13 ± 2.03 (0–8)	1.13 ± 1.15 (0–4)	<0.001 *
14^th^–21^st^ day	1.88 ± 2.33 (0–7)	0 0	<0.001 *

* indicates significant difference (*p* < 0.05).
